# Environmental Determinants of Cardiovascular Diseases Risk Factors: A Qualitative Directed Content Analysis

**DOI:** 10.5812/ircmj.11573

**Published:** 2014-05-05

**Authors:** Leila Sabzmakan, Eesa Mohammadi, Mohammad Ali Morowatisharifabad, Ahmad Afaghi, Mohammad Hassan Naseri, Masoud Mirzaei

**Affiliations:** 1 Department of Health Education & Promotion, Alborz University of Medical Sciences, Karaj, IR Iran; 2 Department of Nursing, Tarbiat Modares University, Tehran, IR Iran; 3 Department of Health Education, Shahid Sadoughi University of Medical Sciences, Yazd, IR Iran; 4 Department of Nutritionist, Qazvin University of Medical Sciences, Qazvin, IR Iran; 5 Department of Surgery, Alborz University of Medical Sciences, Karaj, IR Iran; 6 Department of Surgery, Baqiyatallah University of Medical Sciences, Tehran, IR Iran; 7 Cardiovascular Research Center, Shahid Sadoughi University of Medical Sciences, Yazd, IR Iran

**Keywords:** Environment, Risk Factors, Cardiovascular, Qualitative Research, Precede Model

## Abstract

**Background:**

Cardiovascular diseases (CVDs) are the number one cause of death in the world. In most analyses of health problems, environment plays a significant and modifiable role in causing the problem either directly or indirectly through behavior.

**Objectives:**

This study aims to understand the patients and healthcare providers’ experiences about the environmental determinants of CVD risk factors based on the Precede Model.

**Patients and Methods:**

This qualitative study conducted over six months in 2012 at Diabetes Units of Health Centers associated with Alborz University of Medical Sciences and Health Services which is located in Karaj, Iran. The data were collected based on individual semi-structured interviews with 50 patients and 12 healthcare providers. Data analysis was performed simultaneous with data collection using the content analysis directed method.

**Results:**

Lack of behaviors like stress control, healthy eating and physical activity were the roots of the risk factors for CVD. The environmental factor is one of the barriers for conducting these behaviors. The environmental barriers included of structural environment including “availability and accessibility of health resources”, “new skills”, and “law and policies” which are located in enabling category and social environment including “social support”, “motivation to comply” and “consequences of behavior” which are located in reinforcing category. The most barriers to performing health behaviors were often structural.

**Conclusions:**

The environmental factors were barriers for doing healthy behaviors. These factors need to be considered to design health promotion interventions. Policymakers should not only focus on patients’ education but also should provide specific facilities to enhance economic, social and cultural status.

## 1. Background

Cardiovascular disease (CVDs) are the number one cause of death globally, It is predicted that by 2030, almost 23.6 million people would die from CVDs, mainly from heart disease and stroke (1). In Iran, CVDs are the leading cause of mortality and morbidity, with high cost to health care (2). The Inter Heart study showed that nine modifiable risk factors (abnormal lipids, smoking, hypertension, diabetes, abdominal obesity, psychosocial factors, consumption of fruit and vegetables, regular alcohol consumption and regular physical activity) were associated with more than 90% of the risk of an acute myocardial infarction in this large global case-control study (3). Nonetheless CVDs simplicity and completely are preventive (4). Interventions that have used both educational and environmental measures have been more successful in achieving changes in health behavior than single element interventions (5). A face-lifting environment that makes the health-promoting behavior the easiest behavior to perform is key to a change in the behavior of the at-risk population, as well as to a change of the environmental conditions (6). Examples of environmental conditions include social influences (such as norms, social support, and reinforcement) and structural influences (such as access to resources, organizational climate, and policies). Barriers to performing health behavior are often structural; such as lack of health insurance, high-fat cafeteria foods, high cost of healthy foods, and unsafe neighborhoods for jogging or walking (6). Environmental change usually requires people outside the at-risk population to take action to modify the environmental conditions (6).

To develop effective interventions, it is important to understand the behaviors of target population. Qualitative methods are most ideal for gathering in-depth information to help develop this understanding (7). On the other hand, qualitative methods can help the health educators to more fully understand the health problems, behavioral and environmental causes, and determinants from the perspective of the people involve (8). Several studies have examined environmental and social barriers of healthy behaviors. In this studies the barriers to improve the lifestyle in regards to physical activity and diet included financial , stress (9) lack of social support (10) and cultural barriers, such as lack of women- only exercise facilities (11), unsuitable built environment, unsafe neighborhood and bad weather for doing physical activity and costs of healthy foods, unavailability healthy foods in stores (12, 13). These studies did not use theories related to individual health behavior in order to identify health problems, behavioral and environmental causes and their determinants (14). The PRECEDE model that is often used in health education and health promotion is a logical model that describes the causes of health problem (6). Based on the study by Green, the behavioral causes can be classified to factors as predisposing, enabling and reinforcing. These factors act as determinants for particular behavior, The most effective interventions for improving health can be expanded for each factor (15). The predisposing factors are antecedents to behaviors that include knowledge, attitude, belief, exiting skills, and self-efficacy. The enabling factors are antecedents to behavioral and environmental change include the availability, accessibility, and affordability of health care and community resources, laws and policies. The reinforcing factors are those factors following a behavior that provide continuing reward or incentive for the persistence or repetition of the behavior, which include social support, peer influence, significant others. The planners may see determinants as the processes of change that must be activated or set in motion if the necessary behavioral and environmental changes are to occur (16).

## 2. Objectives

The aim of this study is to understand the patients and healthcare providers’ experiences about the environmental determinants of CVD risk factors based on Precede model.

## 3. Patients and Methods

A qualitative method with directed content analysis approach was selected. The goal of a directed content analysis approach is to validate or extend conceptually a theoretical framework or theory. Existing theory or research can help focus the research question and it can help researchers begin by identifying key concepts or variables as initial coding categories. This approach was employed by Hsieh and Shannon in 2005 (17).

### 3.1. Data Collection

The data were collected through semi-structured in-depth interviews from September to March 2012. After transcription and analysis of each interview, in case of ambiguity and for probing into participants’ experiences, the interview was repeated to clarify different aspects of the subject, if needed. Totally, 62 face-to-face interviews with 50 patients and 12 healthcare providers who work in the Diabetes Units of Health Centers of Alborz University of Medical Sciences and Health Services were conducted. Each interview lasted between 30 to 60 minutes and all the interviews were conducted in a private room. The interview guide consisted of open-ended questions based on subcategories of enabling and reinforcing categories of Precede Model to allow respondents fully explain their own experiences.

### 3.2. Setting and Participants

The average age of participants were 46.5 ± 5.97 (Table 1). The main researcher look at documents of blood test results of patients performed by Diabetes Units’ staff, if patients had at least one biochemical CVD risk factors such as pre-diabetes, type 2 diabetes (T2D), metabolic syndrome, hyperlipidemia and hypertension for at least one month, and previously received advises from healthcare providers to change lifestyle, the main researcher contacted each of the potential participants to explain the objectives and the research questions, and if the participant agreed to take part in the research, an interview was carried out. The patients were chosen using purposive method to consider the maximum variation sampling based on (sex, age, level of education, occupation and the type of risk factor) from four health centers associated with Alborz University of Medical Sciences which is located in Karaj-Iran. The four centers were selected to obtain an understanding of patients with various socio-economic statuses. Patients with previous heart attack, stroke, open heart surgery or angioplasty, newly diagnosed patients (less than one month) and all type 1 diabetes patients were excluded from the study (n = 7). In addition, 12 healthcare providers from the Diabetic Units of four health centers, including three general practitioners, three nurses and three dieticians were individually interviewed. The mean age of healthcare providers was 38.4 ± 9.8 and 10 of them were female.

**Table 1.** The Study Patients’ Characteristics

Variables	Number	Percent
**Sex**		
Female	31	62
Male	19	38
**Educational level**		
Illiterate	5	10
Primary	14	28
High school	16	32
Diploma	7	14
Collage degree	8	16
**Occupation**		
Housewife	29	28
Retired	6	12
Employed	15	30
**Disease**		
Diabetes	28	56
Hyperlipidemia	45	90
Pre-Diabetes	12	24
Metabolic	14	28
Syndrome	10	20

### 3.3. Ethical Considerations

Ethics approval was obtained from Shahid Sadoughi Human Research Ethics Committee (No: P/17/1/57802). Participation was voluntary and written consent was obtained from each respondent. The participants were reassured about confidentiality and anonymity. The authors of this manuscript have certified that they comply with the principles of ethical publishing.

### 3.4. Data Analysis

All interviews were conducted, recorded, transcribed verbatim, reviewed, coded and immediately analyzed by the first researcher. A directed content analysis approach was used for data analysis. According to the directed content analysis process, at first, each interview was read several times carefully to gain deep understanding the data. Coding can begin with one of two strategies, depending on the research question. If the goal of the research is to identify and categorize all instances of a particular phenomenon, such as emotional reactions, then it might be helpful to read the transcript and highlight all text that on first impression appears to represent an emotional reaction. The next step in analysis would be to code all highlighted passages using the predetermined codes (17). Then, important statements were underlined to identify the initial codes or meaning units that exist in the interview text. In the next phase, these similar meaning units (codes) were placed initially in subcategories of the PRECEDE model and then into its three main categories (predisposing, enabling and reinforcing). Any text that could not be categorized with the initial coding scheme would be given a new code. The data collection process was continued until data saturation – when adding further data showed no new information and the extra collected data were redundant. Also, when the new code was not produced in the last four interviews, saturation was achieved and data collection was stopped. When the new code was not produced in the last three interviews, saturation was achieved and data collection was stopped. Because of the large volume of data obtained in this investigation, only enabling and reinforcing categories reported as environmental causes and the predisposing categories will published as behavioral causes as soon as. Placing the codes in subcategories were based on ecological and educational diagnosis phase of PRECEDE model (15). An example of coding and placement in subcategories and categories is shown in table one (Table 2).

### 3.5. Consideration of Rigor

Prolonged engagement in the field from September to March 2012 helped to establish some trust and rapport with participants, providing an opportunity to collect the data. To make sure that the analysis reveals the patients and healthcare providers’ experiences, member checking was performed during the data collection, and where needed, some changes were done. To confirm dependability and conformability of the data, the interviews and results of the analyses, that is, the initial codes, subcategories and the categories of PRECEDE model, were audited by some experts, the external check method using two authors (the first and second author) expertise in health education and familiar with PRECEDE model and correspondence with Professor Green, the designer of PRECEDE model, and peer check by two PhD students in health education who had experience with PRECEDE model were used. Maximum variation of sampling also confirmed the conformability and credibility of data. Sampling strategies allowed for maximum variation to occur and a vast range of views and perspectives to be considered.

**Table 2.** Example of Analysis Process

Meaning Unit	Codes	Subcategory	Category
**“This screening program for diabetes in health centre is useful, and without this facility I didn't know about my disease”**	existence of diabetes unit and having access to members of diabetes team	Availability and accessibility of health resources	Positive Enabling
**“If we want to follow diet, we should afford it. For example, the dietician advised me to consume white meats, but I can't regularly provide that. Fish and olive oil are so expensive”**	high cost follow the diet	laws and policies	Negative Enabling
**“When health workers contact me and ask me to refer to health center, it indicates that they value me”**	support and encourage of health workers	social support	Positive Reinforcing
**“The experience that patients obtain following diet therapy, regular physical activity in controlling weight, blood glucose and lipids encourage them to increase these behaviours”**	positive results and experience from doing health related behaviors	consequences of behavior	Positive Reinforcing

## 4. Results

In this study in category of enabling factors, 37 codes under three subcategories of “availability and accessibility of health resources”; “new skills”; and “laws and policies” were studied. In addition, in category of reinforcing factors, 40 codes under three subcategories of “social support “, “motivation to comply” and “consequences of behavior” were investigated.

### 4.1. Enabling Factors

#### 4.1.1. Availability and Accessibility of Health Resources

In view point of the patients and healthcare providers, the existence of the Diabetes Units and having access to members of the diabetes team (nurse, dietician and general practitioner) were very effective in controlling and awareness of their illness. “This screening program for diabetes in health center is useful, and without this facility I didn't know about my blood glucose and lipids. Especially, being free of charge enabled me to come to health center” (female, 52 aged, diabetes and hyperlipidemia). Both patients and healthcare providers realized the lack of psychologist in the Diabetes Units in health centers, because most of the patients considered the stressful factors as main cause of their illness and that exposing to these factors worsening their situation. Hence, due to that the patients were not able to control the stressful factors, the attendance of psychologist in the Diabetes Units seemed necessary. “Most of the time, I have to give patients psychological advice rather than dietary consultation. In my opinion there is a need for a psychologist to work with us” (Dietician). The patients and healthcare providers realized the existence of referral recording and follow up system in the Diabetes Units. However, they recommended that the mentioned recording and follow up system need to be established for hyperlipidemia, hypertension and pre-diabetes patients too. “Unfortunately, pre-diabetes and hyperlipidemias don't have recording and follow up system. At least in heath, the preventive policy is more import than treatment. We must have recording and follow up system for pre-diabetes to prevent them from diabetes phase, the pre-diabetes need to be covered with diabetes unit” (general practitioner). The diabetes patients and also healthcare providers mentioned the existence of good referral system for preventing complications of diabetes. However their evaluation about the quality and easy accessibility of these services was weak. “Some time we refer the patients to hospital, but they return due to not availability of health professionals and issues of distance and transportation. Hence, the patients are not willing to be referred to hospital for controlling diabetes” (general practitioner). In the Diabetes Units, where there was a specific doctor for diabetes, there was more trust to the doctor than where the head of health center was doing the duty of the diabetes doctor. The patients expressed that having a specific doctor for the Diabetes Unit results in appropriate care and attention to patients. In this case the patients had regular visiting of the Diabetes Unit. However, in the centers where the head of center was doing the Diabetes Unit duties, there was un-satisfaction and irregular visiting of the Diabetes Unit which was confirmed by health providers and specifically doctors. “The only advantage of this screening program was that the patients trust on doctor and continues their treatment. But the disadvantage of the program is the doctors are replaced frequently. The patient says “are you working in this center when I came to next follow up”. The treatment process will be effective when the patient for treatment and follow up trust on his/her doctor” (general practitioner). Lack of separate landline phone in the Diabetes Unit for follow up of patients was one of the problems that most of the healthcare providers and some patients were concerned about it. “Due to not having separate land line phone, we are not able to have easy contact with patients. We cannot contact with their mobile phone and we have to ring their home number. Most of the patients are tenant and they move out after one year. If we had their mobile number, we could easily follow up them” (nurse).

Establishing training courses in order enabling healthcare providers for educating patients was another request of most of the staffs in the Diabetes Unit. Some of the patients also expressed that the educational material of staffs of the Diabetes Unit are not up to date. “The staffs who are working in diabetes unit haven't passed training course. Lack of educational program is a major defect that was observed in the unit and we criticized about that. We need to have up to date educational program running by experts each several months” (general practitioner). Most of the patients mentioned that, they don't have access to booklet and educational pamphlets about their disease, complications of disease, healthy diet, correct consumption of medications, cooking dietary foods and also lack of CDs for exercises. The patients said, in the Diabetes Unit they are given just one page incomplete diet guideline. Most of the patients especially women realized that there are not any gems to be referred by the Diabetes Unit. Some of female patients or their husband mentioned that, there is not any educational program in the Diabetes Unit in relation to cooking methods for their disease. Most of the patients especially pre-diabetic, hypertension and hyperlipidemia explained that, there is not any educational program for them in the health center. This was confirmed by healthcare providers and they told that the educational program is running just for diabetic patients. “The availability of these educational booklets in diabetes unit for diabetic patients is useful and it increases their knowledge. If we receive verbal information, we may forget it. But if this booklet is available, then we can read through and remind it. The dietician gave me some written dietary advice, but I think it was not useful. If the materials to be pre prepared as a booklet, it would be more attractive for reading” (man, 44 aged, diabetic).

“Running educational program in health center about food preparing and diets for diabetic is what we want. There are sometimes television program that I watch and take notes. I wish the health center arranges exercises program and forces us to take part in the programs. It is very good obligation of taking part in programs” (female, 46 aged, hyperlipidemia).

#### 4.1.2. New Skills

Most of the patients felt that they know about healthy foods, but few of them had skills of reading labels of foods and choosing healthy foods when they want to do shopping. Most of them had the problem of calculation of daily energy needs. Some patients also felt that they don't have skill of regular diet programming and some of ladies or their husband believed that they don't have skill of cooking healthy foods or they do over cooking and provide lots of food on table on meal time. Some patients also felt that, they have not skill of walking or exercising programming with someone else and most of them don't have skill of regular walking or exercising programming. “One of the problems is that the housewife has habit of cooking and providing large amount of meal in each plate. The housewife has learned this habit from her mother and is very bad habit” (male, 37 aged, hyperlipidemia).

The ability of most of the patients to control the stress and avoid conflict was completed through deep and meaningful conversation, trusting on God and reciting holy book (Qoran), but none of patients had skill of calmness to control the their stress. “Unfortunately, most of the patients don't have a healthy food even a regular walking program. They don't know even how to control stress” (Dietician).

#### 4.1.3. Laws and Policies

Expenses and financial problems were main issue of the patients and were barriers to follow the diet, physical activity, stress management, referring doctor, and doing libratory tests. The patients and staffs felt that having diet is expensive. They also pointed out to high expense of exercise clubs and swimming pool. “Most of ladies complain from bone pain, osteoporosis, and arthritis. We advise them to do walking in swimming pools, but they don’t do because of costs” (nurse). “If we want to follow diet, we should afford it. For example, the dietician advised me to consume white meats. But, I can't regularly provide that. Fish is expensive. Olive oil is so expensive; a small can is 6000 Tomans. Canola oil is expensive” (female, 43 aged, diabetic). Most of the patients also felt high expense of screening test, doctor visit charges and blood glucose stripe test. “Because our patients have financial problems, I wish either doctor visit charge or laboratory test expense was free of charge. Unfortunately, in admission stage, due to small amount of admission fee, we miss our patients” (nurse). Most of the patients perceived that, the stress is main cause of their illness. They felt that the induced stress due to unemployment, inflation, addiction, divorce, disease and death of family member are main cause and worsening of their illness which are uncontrolled and out of their hand.

“The patients say, the problems I faced resulted in increasing blood glucose, blood lipid, and hypertension. For example they say, my husband or son is unemployed and staying at home. My husband or son is addicted. My daughter is divorced. I have problem with my husband. He doesn't have any feeling towards me. My husband has remarried. Most of the patients point out to these problems as cause of their disease (Dietician). Most of the patients felt in ceremonies and parties the provided foods are high fat and due to soft drinks and sweets, they can't follow diet in these ceremonies. They also felt to availability of prepared and fast foods in the society and willing of families and specially youths for consuming these foods. “Unfortunately, the parties and ceremonies that we attend is a problem. Consuming high fat foods, different types of jelly, and soft drinks that is available in the parties is not avoidable” (male, 49 aged, diabetic and hyperlipidemia). Some of patients also felt to the lack of facilities such as nearby gym, unsafe parks and pedestrian walk ways especially for women, lack of exercise places for women, having car by most the families and bad weather conditions (extreme heat or cold), rainfall or pollution as barriers for exercise or physical activity. Some patients felt that, they don't have access to the shops that selling low fat dairy products, dietary bread and fresh vegetables and they have to walk a long distance to provide these types of foods. “When you are walking, motorcycle and cars suddenly pass next to you causing panic attack. Which cause? In my opinion the streets and pathways are very unsafe places. Few months ago when our neighbor was walking, a car hit to her and her broken leg received internal fixation” (female, aged 41, hyperlipidemia and hypertensive). “Recently, I have put away consuming dietary bread (barely bread), because the shop selling this bread is far away” (man, aged 50, diabetic and hyperlipidemia).

Most of the patients also felt that the unhealthy and low quality foods in the market are one of the factors has role in inducing their risk factors and obesity. They believed, using chemical fertilizers for cultural purpose, food additives, illegal additives, consuming hormonal meats and chickens, and fast foods have increased the chance of getting mentioned disease. Most of the patients felt that the authorities don't supervise the safety of foods. This statement was confirmed by most of the staffs. “Now days, food products contain a lots of chemical substance. Potato has abundant chemical substance. Wheat, and produced bread and fruits contain chemical agent. In the past, these chemical agents were not observed in the foods. Although, chemical fertilizers increase productivities, but cause disease, there is not supervision on food safety and wellbeing of people is not important, they just increase the prices” (man, aged 45, metabolic syndrome).

### 4.2. Reinforcing Factors

#### 4.2.1. Social Support

Most of the patients realized that support and encourage of healthcare providers (doctor, nurse, and dietician), family (spouse, children, and parents), friends and neighbors was effective for conducting regular physical activity, following diet and controlling stress. The patients felt that their motivation reduces when family members don't support them for conducting recommended behaviors. Most of the patients believed that their family just advise them, but don't accompany them for walking and diet. “Children frequently advise me to follow dietary food. My husband eats the dietary food that I provide, but the children don't eat and they say it is steam cook. Family just saying follow the diet, supporting is different from verbal advice” (female, 54 aged, diabetic and hyperlipidemia). Most of the patients felt that they don't have participant to do exercises or walking with them. Some lady patients' husband doesn't allow them to attend gyms and doing walking. “I had walking program with one of my relative for two months, but her husband didn't allow her to join to me anymore, and I was not motivated to continue. It will be encouraging, if two persons to be along with each other for walking program” (female, 46 aged, pre-diabetic and hyperlipidemia). Some patients felt that in the parties they have been forced to eat foods and some patients felt family’s insisting as negative barrier”. Unfortunately, the parties that we take part are a problem. For example, if I don't sit on dining table, they may become upset. They frequently insist for eating” (man, 49 aged, metabolic syndrome).

Most of the patients felt the healthcare providers telephone contacting as a positive point in encouraging and follow up of their disease. They also perceived that being free charge of blood test for screening purpose encouraged them to refer to the Diabetes Unit of health centers. “This follow up program is very good. When they contact me and ask me to refer to health center, it indicates that they value me. Free charge blood test also is encouraging for going to clinic” (man, diabetic). The patients felt that, the healthcare provider’s behavior and their good communication was important factor for considering their advice and next referrals. They felt that, if the doctor be kind and allocate enough time for visiting patients and consultation, the patients follow the given advices and referrals. But most of the patients said, doctors just prescribe medication and they don't do consultation. The doctors have less attention to the patients’ diet program, stress control, and physical activity program. However, the patients realized that the role of dietician and nurse in consulting with them is very useful”. When the patient is given good educational program and doctor allocate enough time for consultation, the patient will have more connection and referrals. The relation of doctor and patient is important. The way doctor approaches and his/her explanation to patient; effect on how seriously the disease to be considered by patients” (doctor).

#### 4.2.2. Motivation to Comply

Most of the patients especially patients suffering from pre-diabetes, hypertension and mild hyperlipidemia (triglyceride and cholesterol lower than 300 mg/dL) felt that, their family (spouse, children, parents) and the healthcare provider’s (doctor, dietician, nurse) advices for following diet, controlling stress and doing regular physical activity are not very important and they don't follow their advices. Of course, majority of patients who use medication realized that, their doctor's prescription is important and follow the doctor's medication order. But they don't listen to the diet, stress control and regular physical activity advices. “My husband and my mother frequently ask me avoid eating those foods that are harmful for me and intake my medication, but I don't care” (female, aged 39, hyperlipidemia). “Unfortunately, our nation is drug consumer. They consider consuming drug so serious, but not diet, regular physical activity, and so on” (Dietician).

#### 4.2.3. Consequences of Behavior

The patients who have obtained positive results and experiences (such as weight loss, blood glucose and hyperlipidemia control; hypertension control, feeling good, being fit, and disappearance of disease sign) from following die, physical activity and stress control would encourage to continue these behaviors. “The experience, they obtain following one month diet therapy and regular physical activity in controlling weight, blood glucose and lipids encourage them to increase these behaviors” (Dietician). Some patients felt that when they have stress, they eat a lot and enjoy from eating foods. In fact they think, the stress is a factor which leads to worsening their illness. “When I become angry, my blood glucose, and blood pressure elevate, and sweating increases. I experienced that the stress increases my appetite” (man, aged 49, metabolic syndrome). Some patients, who were feeling unwell and were experiencing pain during exercise, stopped taking part in physical activity, “If I do walking, my back and legs pain develops and I stop the activity” (female, aged 44, hyperlipidemia).

## 5. Discussion

The experience of patients and healthcare providers showed that, lack of behaviors such as stress control, healthy diet and physical activity were the main cause of the risk factors of CVD. They believed that the environmental factor is one of the barriers for conducting these behaviors. In subject’s view point, the environmental barriers included of structural and social environment. The structural factors are in three subcategories of “availability and accessibility of health resources”, “new skills”, and “law and policies” which are located in enabling category. The social factor included of three subcategories of “social support“, “motivation to comply” and “ consequences of behavior” which are located in reinforcing category. In the view points of patients and health care providers, the most barriers to performing healthy behaviors were often structural (i.e. enabling factors). As mentioned, in most analyses of health problems, the environment plays a significant and modifiable role in causing the problem either directly, or indirectly through behavior (6). The results of this study also confirmed the indirect effects of environmental factors (i.e. environmental barriers) on conducting behaviors related to control of CVD risk factor. The results of current study demonstrated that, the availability of the Diabetes Unit and free of charge blood test in health centers resulted in awareness of patients about their disease. The existence of dietician, nurse and general practitioner in the Diabetes Unit has main role in increasing awareness of patients and controlling their disease. However, the patients and healthcare providers pointed out to the low quality of health services, shortage of expert human resource and facilities in the Diabetes Unit. In accord with the findings of the studies by Shakibazadeh et al. (18), Crasson et al. (19), Zgibor et al. (20) and Cook et al. (21), health care systems barriers are one of the major reasons for not controlling CVD risk factors. The most patients and healthcare providers mentioned that, financial difficulties, health care costs, health insurance coverage, transportation, time, insufficient access to a multidisciplinary team and difficulties getting appointments and referrals are barriers of healthcare systems. Subjects mentioned that, health system-related barriers that limited either the healthcare providers’ ability to deliver care or patients' ability to access appropriate care. Patients with limited economic resources were unable to adopt the complex medical and lifestyle regimens required for good CVD risk factor control. In this study, healthcare providers mentioned that, a barrier is not only access to any care, but also access to quality care. In regarding to high costs of health care and importance of role primary health care in expansion equity in healthy systems, it recommends the Screening Unit replaced the Diabetes Unit for controlling non-communicable diseases and access to a multidisciplinary team to provide care for patients in order to decrease unhealthy behaviors and controlling CVD risk factors, and finally to prevent of CVD In current study, one of barriers prevented patients from achieving health related behaviors was, not have sufficient skills in behaviors such as healthy diet, appropriate physical activity and stress management. Study by King et al. (22) revealed that health professionals play a crucial role in patient education but rarely receive training in effective teaching and counseling techniques. The effective patient teaching and problem solving course improved several kinds of important skills and resulted in improved glycemic control, as health providers attempt not just to teach but also to help patients overcome considerable obstacles to consistent diabetes self-management. In our study, also the healthcare providers mentioned to the lack of continuing professional development programs such as awareness of up to date findings, about control of risk factors and principle of appropriate educational programs to patients. One of the barriers, which most of the patients and healthcare providers mentioned, was the unavailability of psychologist in the Diabetes Unit. Most of the patients felt that, the stress of living situation such as unemployment, addiction, divorce, financial difficulties and family problems are main cause of their illness. Hence, availability of a psychologist in screening unit for consultation with the patients to control their stress and helping them cope with life difficulties seems necessary. In concurrent with our findings, Schulz (23), Nakkash (9), and Farooqi (11) in their studies revealed that the patients mentioned that, their stressful life situation such as stress related to job, financial, crime and unsafety of living environment are main causes of their disease. Similarly, the patients in our current study explained that, in the stressful life situation, they are not able to control their blood sugar, lipid and blood pressure, which could be due to the indirect effect of stress on diet, physical activity, and correct consumption of medication. Studies (24) demonstrated that stress indirectly impact on coronary heart disease by affecting behavior such as low physical activity and poor diet.

The patients in this study also realized that, one of very important barriers for following healthy diet, regular physical activity were the financial problems, they mentioned to the high cost of healthy food and high cost of swimming pools and gym as a barrier. These findings have been confirmed by other studies conducted by Nakash (9), Chow (12), and Popkin (13). In the current study, the inappropriate built environment was another barrier preventing patients to follow the diet and to do the regular physical activities. In regard to following healthy diet, some patients felt that they don't have access to fresh and healthy foods with high quality and they realized that, the food additives and using fertilizers in plants is the main cause of their disease. They also realized that, there is not healthy food policies for controlling food quality and marketing junk foods. Some employed patients realized that the unavailability of healthy foods in work place is main factor for not following diet. In addition, they mentioned that availability of fast foods restaurants in the cities and willingness of family members, especially youths for eating outside is the reason for obesity, CVD risk factors and possibility of CVD in the future. These finding were similar to the results of studies conducted by Chow (12), Story (25), Rabinson (26) and Popkin (13). The effect of physical environment on nutritional behaviors and fruit and vegetable consumption among African American has been reported by Rabinson (26). This report demonstrated that, the access to fresh and healthier food in shops and supermarkets nearby living places increases subjects' motivation for providing these foods and low income households may not be able to make such trips to farther stores. The study of Story (25), explains that, the physical environment includes the multiple settings where people eat or procure food such as the home, work sites, schools, restaurants, and supermarkets. The physical settings within the community influence which foods are available to eat and impact barriers and opportunities that facilitate or hinder healthy eating. Macro level environmental factors play a more distal and indirect role but have a substantial and powerful effect on what people eat. Macro-level factors operating within the larger society include food marketing, social norms, food production and distribution systems, agriculture policies, and economic price structures.

Finding of this study showed, built environment impacted on physical activity. In the viewpoint of the patients, especially presence of crime in the parks and neighborhoods, and unsafely in footpaths were important barriers for women's walking. They also, realized that, using private cars, very cold or hot weather, and air pollution are other important factors prevent them to do physical activity especially walking. Some patients expressed that, inaccessibility of gyms and travelling cost to recreational areas (gyms and sporting centers) are main barriers for preventing them to do physical activity. Similar findings were reported by Oliveria-Brochaed (27), Nakkash (9), Shibata (28), Chow (12), and Sjostrom (29). However, the difference between these findings and our findings was that most of the women in this study due to cultural issues such as veil and limitations that they were experiencing were not able to use fitness equipment in the parks; some women were also embarrassed of exercising in public places which men are watching them. In addition, the absence of appropriate specific sporting fields for women in the cities was felt by most of the females’ patients in our study. In the study conducted by Shabita (28) and Cerin (30), the neighborhood attractiveness and having fitness equipment at home were factors affecting on improvement of physical activity. Although, the patients in our study didn't feel that these factors effect on their physical activities, but probably, this was due to that, our patients didn't have fitness equipment because of their financial problems and high cost of fitness equipment’s. However, the patients didn't mention the availability of low cost fitness equipment at home; this shows that physical activity is not valued for most of our studied patients. According to the definition of Stahl and Boman (5), environment can be seen as the extent to which there are incentives or restrictions that make health behavior easier or less easy. Incentive environments are those which provide best access to facilities for physical activity such as sport fields, bicycle paths and swimming pools. Restricting environments are those which con-strain access, or provide attractive sedentary environments, for example busy highway systems, sedentary games rooms and many office-based workplaces. Strongly related to these environments are the regulations and policies that create opportunities or restrictions for physical activity are still quite limited. Social relationship and social support are one of the key elements of the social environment that influence on healthy behaviors and health status. In our current study, social environment was found as one of the factors affecting on following diet, stress control and physical activity. Family support (spouse, children and parents), advices and encouragement of health providers (general practitioner, nurse, and dietician) and friends’ recommendation have important role for starting and continuing these behaviors. In the study carried out by Goetz (31), the role of the social support of general practitioners and nurses for changing diet and physical activity for people with type 2 diabetes has been reported. The other studies such as Oliveria-Brochado (27), Stahl (5), Trost (32), Cerin (30) and Shabita (28) also demonstrated that social support from family, friends, peers and doctors advices have positive effect on physical activity. In addition, Sallis (33) explained that, the influence of social support on physical activity could be direct (such as exercising together or taking care of children for the spouse to exercise) or indirect (as encouraging a friend or family to be more active) and the preferred type of social support varies according to gender and age group. Moreover, Robinson (26) reported that interpersonal factors also played a key role in the dietary behaviors of Africans American. The interpersonal level of influence (interpersonal processes, and primary groups including family, friends, peers, that provide social identity, support and role definition) may also be viewed as the individual‘s social environment. Cultural traditions and role expectations are considered as important factors. Also the role of women was a major focus of the interpersonal processes influencing dietary behaviors, because they were primarily responsible for shopping and preparation of food, they are significant sources of health and nutrition information, and they were usually more interested in improving their health habits. Story (25) reported that, the social environment includes interactions with family, friends, peers, and others in the community and may impact food choices through mechanisms such as role modeling, social support, and social norms. The results of current study showed that, the behavioral advices such as physical activity, healthy diet and stress control which were recommended by general practitioner, dietician, and nurse were less acceptable by patients. But, the patients especially those with low socio-economic and education levels had more attention to doctors' medication order. The preference in acceptance of doctor's medication order in compare to acceptance of other behavioral advice was confirmed in reports of Mishali (34) and Zgibor (20). The negative or positive experiences of individual from behavior were one of the supportive factors in our current study that influenced on doing or not doing healthy related behaviors. Reinforcing factors are those consequences of action that determine whether the actor receives positive (or negative) feedback and is supported socially afterward (15). As our finding demonstrated, the patients with controlling stress, doing physical activity and following diet had good feeling. Experience of controlling blood sugar, lipids and blood pressure resulted in to be encouraged to continue these behaviors. Shabita (28) reported that, the feeling of pleasure of a physical activity is a supportive factor and study of Parschau (35) showed that, participants' perceptions of positive experience were associated with their subsequent self-efficacy fostering physical activity. In this regard, Shabita (28) and Sjostrom (29) mentioned that, the frequent watching of individuals' exercises is a supportive factor for doing physical activity. Displaying programs for prevention from CVD on TV through short programs at peak hours that causes a lot of people would see them, distributing flyers door-to-door, advertising, establishing community activities such as lectures in relation to preventive behaviors of CVD in schools targeted parents, in workplaces and in health centers, establishing exhibitions for teaching accurate cooking skills to women and providing classes for teaching relaxation in order to stress control, establishing walking congress and also affordable gyms in health centers and encouraging and following patients by healthcare providers for taking part in mentioned activities were methods as perceived by patients and healthcare providers in order to reducing CVD risk factors, and finally to prevent of CVD which were similar to finding of Nakkash (9). Limitations: This study was a qualitative study and the findings can provide a deep understanding of the environmental factors for conducting health related behaviors that could not be achieved through quantitative study, but the study was carried out by patients who had sought care in a the Diabetes Unit of Karaj Health Centers which may not be representative of the general population of the patients with CVD risk factors who may refer to private clinics etc. This influences the applicability of the findings. Despite these limitations, variety in sampling was an advantage of the current study. Participants of the study belonged to different socio-economic backgrounds, were of various degrees of CVD risk factors, and were of both genders and the variety of ages, it increase applicability of findings. Also findings of current study could support the subcategories of predisposing category of PREEDE model, Moreover data was analyzed appropriately and results were corroborated by using multiple reviewers, especially correspondence with professor Green, the designer of PRECEDE model, to ensure that participants' viewpoints were adequately interpreted.

In subject‘s view point, lack of behaviors such as stress control, diet therapy and physical activity were the main cause of the risk factors of CVD and the environmental factor is one of the barriers for conducting these behaviors. The environmental barriers included of structural and social environment. The structural factors are in three subcategories of “availability and accessibility of health resources”, “new skills”, and “law and policies” which are located in enabling category. The social factor included of three subcategories of “social support”, “motivation to comply” and “consequences of behavior” which are located in reinforcing category, that the most barriers to performing health behavior were often structural (enabling factors). The environmental factors are barriers for doing health related behaviors, it recommends in order to designing health promotion programs, policymakers not only focus on patients' education but also consider specific facilities in related to economic, social and cultural status. Then Identifying these determinants will help the program planners in designing of future programs to select the most appropriate methods and applications to address these determinants in order to decrease unhealthy behaviors in order to reducing CVD risk factors, and finally to prevent of CVD, because the most immediate impact of an intervention is usually on a set of well-defined determinants of behavior.

## Appendix:

The interview guide consisted of open-ended questions based on subcategories of enabling and reinforcing categories of PRECEDE Model:

**A: Questions to be Asked From Patients:**

1. Tell me about your experiences of using the Diabetes Units services in terms of managing your illness.
2. Tell me your experiences about role of healthcare providers (doctor, nurse and dietician) for doing physical activity/ following diet and controlling stress.
3. Tell me your experiences about role of family for doing physical activity/ following diet and controlling stress.
4. Tell me your experiences about environmental barriers for doing physical activity/ following diet and controlling stress.
5. Tell me about your abilities and skills for doing regular physical activity/ following diet and controlling stress.
6. Tell me about your feelings after doing regular physical activity/healthy diet and controlling stress? (positive and negative consequences)
7. What problems have you experienced in the Diabetes Units?
8. Is there anything else important you would like to talk about?

**B: Questions to Be Asked From Healthcare Providers:**

1. What is role of the Diabetes Units in managing patients’ illness?
2. How do you influence your patients to do physical activity/ to follow a diet and to control stress?
3. How do family influence your patients to do physical activity/ to follow a diet and to control stress?
4. How are patients’ abilities and skills for doing physical activity/ following diet and controlling stress?
5. What are environmental barriers for doing physical activity/ following diet and controlling stress?
6. What motives patients to do physical activity/ to follow a diet and to control stress?
7. What problems have you experienced in the Diabetes Units?
8. Is there anything else important you would like to talk about?
